# In Vitro Study of the Antimicrobial and Prebiotic Activity of *Origanum heracleoticum* Hydrolates from Two Italian Crops and the Proteomic Response of Probiotic Bacteria

**DOI:** 10.3390/biology15201857

**Published:** 2026-10-15

**Authors:** Silvia Rizzo, Filippo Schiavone Panni, Maura Di Vito, Stefania Garzoli, Domiziana Coggiatti, Melinda Mariotti, Maurizio Sanguinetti, Anna Percio, Andrea Urbani, Viviana Greco, Francesca Bugli

**Affiliations:** 1 Dipartimento di Scienze Biotecnologiche di Base, Cliniche Intensivologiche e Perioperatorie, Università Cattolica del Sacro Cuore, Largo F. Vito 1, 00168 Rome, Italy; silvia.rizzo@unicatt.it (S.R.); filipposchiavonepanni@gmail.com (F.S.P.); domiziana.coggiatti@unicatt.it (D.C.); melinda.mariotti@unicatt.it (M.M.); maurizio.sanguinetti@unicatt.it (M.S.); anna.percio@unicatt.it (A.P.); andrea.urbani@unicatt.it (A.U.); viviana.greco@unicatt.it (V.G.); francesca.bugli@unicatt.it (F.B.); 2 Dipartimento di Scienze di Laboratorio e Infettivologiche, Fondazione Policlinico Universitario “A. Gemelli” IRCCS, Largo A. Gemelli 8, 00168 Rome, Italy; 3 Dipartimento di Chimica e Tecnologie del Farmaco, Università di Roma Sapienza, Piazzale A. Moro 5, 00185 Rome, Italy; stefania.garzoli@uniroma1.it

**Keywords:** essential oil, *L. rhamnosus*, *E. coli*, steam distillation, Caco-2

## Abstract

This study explores the chemical composition and biological activities of essential oils (EOs) and hydrolates (Hys) from *Origanum heracleoticum* (OH), an aromatic plant with known applications in the food industry. While EOs have been widely studied, Hys (co-products of EO distillation) remain largely overlooked. OH was cultivated in the Lucanian and Casola areas, respectively in the regions of Basilicata and Emilia-Romagna, to evaluate the influence of geographic and climatic factors on product composition and activity. Gas chromatographic analysis showed significant variability between the EO and its related Hy. Carvacrol emerged as the dominant compound, ranging from 71% to 91% in both extracts followed by γ-terpinene and thymol in EOs and Hys, respectively. Both EOs and Hys exhibited antimicrobial activity against tested bacterial strains even if active compounds of Hys were relatively more effective than that of EOs. Notably, *Lactobacillus rhamnosus* (probiotic) showed greater resistance to EOs compared to *Escherichia coli* (pathogenic), whereas Hys inhibited *L. rhamnosus* at 25% *v*/*v*, while no MIC against *E. coli* was reached within the tested concentration range (>50% *v*/*v*). Interestingly, sub-inhibitory concentrations of Hys (especially those from Lucan area) stimulated *L. rhamnosus* growth, promoting earlier exponential phase entry and higher final cell densities. Proteomic analysis revealed associated changes in protein secretion, suggesting adaptive metabolic responses. These findings highlight the distinct chemical and biological properties of Hys obtained from a spice widely used in food, and their potential as functional agents. Their ability to influence *L. rhamnosus* growth in vitro supports further investigation of their potential microbiota-modulating properties and the development of food supplements and microbiome-friendly formulations.

## Simple Summary

Essential oils obtained from aromatic plants are widely used in foods, cosmetics, and health-related products, but much less attention has been paid to hydrolates, the water-based co-products generated during essential oil distillation. Essential oils and hydrolates obtained from *Origanum heracleoticum*, a culinary and aromatic plant grown in two different Italian regions, were investigated to understand how growing conditions influence their composition and biological properties. Differences in the chemical composition of the two products were observed, and both were found to limit the growth of the tested bacteria. Interestingly, hydrolates showed biological activities that were distinct from those of essential oils. While they inhibited some bacteria, low concentrations also stimulated the in vitro growth of beneficial microorganisms that are commonly associated with gut health. Changes in proteins released by these beneficial bacteria suggested that they were adapting their metabolism to the presence of the hydrolates. These findings highlight the value of hydrolates, which are often overlooked despite being natural products derived from plants already used in food production. Their potential to support beneficial microorganisms opens new opportunities for developing food supplements and other products designed to promote a healthy microbial balance.

## 1. Introduction

Oregano is one of the most used aromatic plants in the Mediterranean region especially in Southern Italy, where it is widely used in food seasoning. The genus *Origanum* (*Lamiaceae*) includes about 70 taxa, including species, subspecies and hybrids native and widespread throughout the Mediterranean area [1]. *Origanum vulgare* subsp. *viridae* (also known as *Origanum heracleoticum*, OH) and *Origanum vulgare* subsp. *hirtum* (known as Greek oregano) are the most widespread subspecies in Italian regions. The two subspecies are distinguished by small morphological traits such as the density of sessile glands which are greater in subsp *hirtum* than in subsp *viridae*. Due to its GRAS status and high essential oil yield, OH is widely used in agriculture, veterinary practice, and food packaging for microbial control and shelf-life extension. OH is an aromatic medicinal plant studied in ethnomedicine for its antimicrobial [2] and antioxidant [3] properties but also in ethnoanthropological sciences (which describe aspects of the peasant and pastoral economy) [4], for unpublished and now lost popular uses. Its biological activity is largely associated with the volatile fraction of the plant, particularly the essential oil (EO), whose composition may vary considerably according to plant origin and growing conditions. Previous studies have demonstrated substantial variability in the chemical composition of OH-EOs from different Italian geographical areas. A recent study by Napoli and colleagues describes the chemical composition of EOs extracted from three different Sicilian crops (Italy) in three consecutive years. The phytocomplex of EO extracted from this ecotype presents a high concentration of thymol (about 50%) and a lower concentration of carvacrol (<1%) [1]. In contrast, Amato et al. also analyzed the chemical composition of two EOs obtained from two different OH ecotypes in Cilento (Italy). However, the EOs extracted from these ecotypes have a different chemical composition than those shown by Napoli et al. Specifically, these ecotypes have a higher concentration of carvacrol (about 50%) than thymol (<1%) [5]. These differences indicate that the volatile profile of OH may vary markedly among geographical origins and support further investigation of plants cultivated under different environmental conditions. In addition to their chemical variability, OH-EOs have shown antimicrobial activity against different microorganisms [2,6,7], supporting interest in their potential biological applications.

As is known, through the hydrodistillation method, not only the EO but a co-product known as hydrolate (Hy) is also obtained. In contrast to EOs which are characterized by hydrophobic terpene molecules, Hys are hydrophilic solutions characterized by a maximum of 1 g/L of the terpene components of the corresponding EO [8]. A previous study by Di Vito et al. demonstrated that the volatile substances of Hys are relatively more effective than those of EOs, as to obtain a 50% of growth inhibition of a microbial strain, a higher concentration of volatile chemicals is necessary in EOs than in Hys [9]. However, compared with EOs, the chemical and biological properties of OH remain less extensively characterized.

Despite the strong interest in the use of OH-EO in the food industry, information on the biological properties of OH-Hys remains limited, particularly regarding the relationship between their chemical composition and its antimicrobial activity and potential impact on probiotic strains often used as food supplements to support antibiotic therapies. Moreover, little is known about whether sub-inhibitory concentrations of these hydrolates can modify the growth or protein-expression profile of probiotic bacteria. In this study, starting from the seeds of an OH plant propagated since 1960 in the “Giardino delle erbe” of Casola Valsenio (RA, Italy), two OH crops were made simultaneously in the Reggio Emilia region (in Northern Italy) and Basilicata (in Southern Italy). This study aims to (i) evaluate the chemical variability of EO and Hy obtained from two crops of OH collected at its balsamic period; (ii) study the antimicrobial activity of both EO and Hy against a pathogenic and a probiotic strain, *Escherichia coli* and *Lactobacillus rhamnosus*, respectively; (iii) evaluate the influence of Hy on the protein pattern of the probiotic strain; and (iv) assess the effects of increasing HY concentrations on Caco-2 cell viability.

## 2. Materials and Methods

### 2.1. Cultivation of the Officinal Plants

The seeds used to obtain the OH plants, a subspecies of *Origanum* vulgare, were self-produced by “Il Giardino delle erbe” in Casola Valsenio starting from mother plants of Sicilian origin from the early 1960s; the botanical identity of this oregano species was subsequently verified by consulting LaRAS (Seed Research and Testing Laboratory; University of Bologna), a laboratory dedicated to the study, quality control, and certification of seeds. The same seed lot was used for the cultivation of plants in both experimental sites, thus minimizing potential variability related to genetic background or ecotype. These were put in 3 containers with 84 plastic cells each, containing soil and maintained in a greenhouse (23 °C, 16 h of photoperiodic light) with irrigation as needed. The process was carried out simultaneously in the southern part of Italy within the farm “Azienda Agricola San Germano” (PZ, Italy) (40.8026514, 15.9882188) and in the northern part of Italy within “il Giardino delle erbe” (RA, Italy) (44.2310614, 11.6242738) on 20 May 2022, in order to have the procedures as similar as possible. This was a crucial point for the study since the major aim was to explore how the differences in environment, weather and soil would have affected the distillation. Four weeks after sowing, when the two cotyledons that characterize the embryo were completely expanded, the experimental plants were selected and planted in shaded soil in the two agricultural experimental fields. Each field in the two locations consisted of at least 50 oregano plants. To prepare fresh material (top leaves and inflorescences) for distillation, an average of 20 selected mature oregano plants were collected and pooled together to form a single bulk sample of 0.8 kg (average biomass harvested per plant: by dividing the total yield of 0.8 kg by the total number of collected plants, approximately 20, the resulting average is 40 g/plant). Care was taken to ensure that the experimental plants were as uniform in size as possible. During the cultivation period, plants did not receive any treatment and were irrigated twice daily (morning and evening) during the first 10 days and once daily throughout the subsequent days. Each irrigation event lasted 30 min. This irrigation regime was adopted to standardize water availability across all plants and reduce variability attributable to differences in water supply. The soil of the two locations was almost similar: sandy clay loam (60% sand, 20% silt and 30% clay) with ca. 1.5% organic matter, pH of 7.2. Meteorological conditions were evaluated over the period from 20 May to 15 July 2022, corresponding to the interval between planting and the onset of flowering. During this period, Casola Valsenio showed a mean air temperature of 23.6 °C, with mean daily minimum and maximum temperatures of 17.4 and 29.1 °C, respectively, and a cumulative precipitation of 102 mm. At the Acerenza site, the mean air temperature was 22.2 °C, with mean daily minimum and maximum temperatures of 17.0 and 27.8 °C, respectively, and a cumulative precipitation of 96 mm. Mean relative humidity was 58.3% in Casola Valsenio and 52.1% in Acerenza. These parameters were used to characterize the environmental conditions experienced by the plants during the cultivation period.

After 149 days, manual harvesting was carried out in the agricultural experimental fields of the “Azienda agricola San Germano” and in the “Giardino delle Erbe” followed by simultaneous distillation, in the afternoon of the same day, of the two samples of plant material in Casola Valsenio. For the collection, each plant was cut at approximately 20 cm from the ground, but a careful visual selection was performed during harvest. Only the parts containing leaves and flowers were retained for distillation, while the thick, non-secretory main stems were excluded. This visual selection successfully standardized the biomass composition between the Lucan and Casola locations, ruling out any morphological bias in the essential oil yield and composition. The collection was made in each location during the summer phase (between late June and August), when oregano plants reached their maximum concentration of OE EO and peak fragrance in the flowers. In fact, the ‘balsamic period’ of OH coincides with the beginning of its flowering stage. To determine if the oregano plants of the two locations had reached their ideal balsamic period, the ‘Flowering Stage’ (the main indicator) was observed: when the first small flowers begin to open on the flower heads and the flower buds are fully formed, with just the first few blossoms opening.

### 2.2. Distillation 

The EOs and Hys were produced by steam distillation for 1.5 h of 0.8 kg of oregano fresh mass. One EO extraction was carried out using a Clevenger-type apparatus (Albrigi Tecnologie, Verona, Italy) according to the procedures described in the European Pharmacopoeia. Hydrodistillation was performed using a plant material-to-water ratio of 1:12.5 (*w*/*v*) (corresponding to 0.8 kg of fresh plant material in 10 L of distilled water). The EO yield was calculated by dividing the volume of extracted EO obtained by the weight of the starting fresh plant material, then multiplying the result by 100. The distiller used was built by the University of Modena in the late 1970s. It had a modified Clevenger-type apparatus where the insertion of a small glass tube in the cooling coil exploits the principle of communicating vessels and brings the aromatic waters back into circulation to be redistilled again. This ensures that the aromatic waters are more concentrated than those that are normally distilled with steam distillation. Furthermore, this method allows less water to be used in the distillation process, creating a closed cycle. At the end of the process, the EOs were separated from the aromatic waters by decantation. To separate the residual fraction of essential oil dispersed as microdroplets, the hydrolate was subjected to centrifugation at 3500 rpm (approximately 1500× *g*) for 20 min at room temperature. This procedure allowed the removal of any remaining undissolved essential oil, yielding a clear and homogeneous hydrolate suitable for subsequent biological assays. At the end of the distillation process, the EOs and Hys were collected by decantation in sterile amber glass containers and immediately submitted for quality and microbiological analyses. Transport and subsequent storage were carried out at +4 °C.

### 2.3. Headspace Solid-Phase Microextraction Coupled with Gas Chromatography–Mass Spectrometry (HS-SPME-GC/MS) Sampling

To describe the volatile chemical profile of the Hys, the SPME sampling technique was used. About 2 mL of each sample was placed inside a 7 mL glass vial with a polytetrafluoroethylene (PTFE)-coated silicone septum. To collect the volatiles in the adsorption phase, an SPME device from Supelco (Bellefonte, PA, USA) with 1 cm fiber coated with 50/30 μm DVB/CAR/PDMS (divinylbenzene/carboxen/polydimethylsiloxane) was used. Before use, the fiber was conditioned at 270 °C for 30 min. After achieving equilibration, obtained by heating to a suitable temperature and time, the fiber was exposed to the headspace of the samples for 30 min at 40 °C to capture and concentrate the components. Lastly, the SPME fiber was inserted in the GC injector maintained at 250 °C in split mode for desorption of the compounds.

### 2.4. GC-MS Analysis

To chemically characterize the Hys and Eos, the analyses were carried out on Clarus 500 model Perkin Elmer (Waltham, MA, USA) gas chromatograph coupled with a mass spectrometer equipped with an FID (flame ionization detector). The chosen capillary column was a Varian Factor Four VF-1. The GC oven programmed temperature was set initially at 60 °C and then increased to 220 °C at 6°/min and finally held for 20 min. Helium was used as carrier gas at a constant rate of 1 mL/min. MS detection was performed with electron ionization (EI) at 70 eV operating in the full-scan acquisition mode in the *m*/*z* range 35–450 amu (atomic mass unit). The identification of compounds was performed by the comparison of the MS-fragmentation pattern of the analytes with those of pure components stored in the Wiley 2.2 and Nist 11 mass spectra libraries database. Additionally, the Linear Retention Indices (LRIs) were calculated using a series of alkane standards (C8–C25 n-alkanes). The obtained LRIs were compared with available retention data reported in the literature. The relative amounts of the components were expressed as percent peak area relative to total peak area without the use of an internal standard and any factor correction. All analyses were carried out in triplicate.

### 2.5. Bacterial Strains and Growth Media

The antimicrobial activity of the natural products was evaluated against a single clinical isolate of *E. coli* (designated as AEC.51 in the strain collection) and *L. rhamnosus* DSM 20021. The *E. coli* isolate was recovered from a positive blood culture collection of the Fondazione Policlinico Universitario Agostino Gemelli IRCCS (Rome, Italy). Species identification was performed by MALDI-TOF MS, while antimicrobial susceptibility testing was carried out using the VITEK 2 system and interpreted according to EUCAST criteria. The isolate exhibited a susceptible phenotype to the antimicrobial agents tested. LB broth (LLG-Labware Meckenheim, Colonia, Germany) and MacConkey agar (Biolife, Milan, Italy) were used to grow *E. coli* at 37 °C for 24 h. LB broth (LLG-Labware Meckenheim, Colonia, Germany) and LB agar (LLG-Labware, Meckenheim, Colonia, Germany) were used to grow *L. rhamnosus* at 37 °C for 24 h.

### 2.6. Broth Microdilution Susceptibility Test on OH-EOs and OH-Hys

According to the European Committee on Antimicrobial Susceptibility Testing (EUCAST) international guidelines, broth microdilution susceptibility tests were performed to evaluate minimum inhibitory concentration (MIC) and minimum bactericidal concentration (MBC) of OH-EO. Briefly, using 96-well plates, 50 μL of a cell suspension equal to 1 × 10^6^ UFC/mL was added to 50 μL of broth in which a known concentration of EO was previously suspended. The final concentrations of the natural products in each well ranged from 1% to 0.0156% (*v*/*v*) for EOs (0.05% *v*/*v* of Tween 80 was utilized as emulsifier) and from 50% to 0.37% (*v*/*v*) for Hys. The plates were incubated for 24 h at 37 °C. Growth controls were included in each experiment. MIC values were visually determined. The MIC was defined as the lowest concentration with total inhibition of growth compared with growth control. The MBC was evaluated by seeding and incubating 3 μL of the content of each well on MacConkey agar plates or LB agar plates at 37 °C for 24 h. The MBC was defined as the lowest concentration with the death of at least 99.9% of the initial inoculum. All tests were performed in triplicate.

### 2.7. Growth Kinetics Test on OH-EOs and OH-Hys

The analysis of Hys activity on bacteria growth was carried out measuring the absorbance at 630 nm every half hour for 24 h using the Cytation 5 Cell Imaging MultiMode Reader (Biotek, Winooski, VT, USA). Concentrations ranging from 50% *v*/*v* to 0.37% *v*/*v* of the two Hys were tested in 96-well multiwell plates, set up for MIC evaluation, and incubated in the instrument at 37 °C under continuous orbital agitation (205 cycles per minute). For *L. rhamnosus* strains, incubation was performed in an atmosphere containing 5% CO_2_. Data acquisition and growth-curve analysis were performed using Gen5 software (version 3.x, BioTek Instruments, Winooski, VT, USA).

### 2.8. Proteomic Analysis

Cell pellets were obtained from *L. rhamnosus* cell cultures, grown under three different conditions. A 3% *v*/*v* solution of Lucan-Hy and 3% *v*/*v* solution of Casola-Hy have been respectively added to two separate conditions, whereas the third condition, comprising LR alone, was used as control.

#### 2.8.1. Sample Preparation for Mass Spectrometry Analysis

To study the cellular proteome, for each experimental condition, cell pellets obtained from three independent experiments were pooled prior to protein extraction, generating one pooled sample for each condition (*L. rhamnosus* control, *L. rhamnosus* R + Lucan-Hy, and *L. rhamnosus* + Casola-Hy). The pooled cell pellets were then subjected to protein extraction using RIPA lysis buffer (150 mM NaCl, 1 mM EDTA, 1% NP40, 0.5% sodium deoxycholate, and 0.1% sodium dodecyl sulphate [SDS], Sigma-Aldrich, St. Louis, MO, USA) containing a protease inhibitor cocktail (Sigma-Aldrich). Each whole-cell protein extract was subjected to protein digestion according to the Filter-Aided Sample Preparation (FASP) protocol described by Distler et al.. Briefly, 50 µg of proteins from each pooled sample was reduced (DTT 8 mM in 8 M urea buffer and 100 mM Trizma Base), alkylated (IAA 50 mM in 8 M urea buffer and 100 mM Trizma Base) and then digested with trypsin (final trypsin concentration of 0.01 μg/μL) overnight at 37 °C on Microcon Centrifugal Filter Devices (Merck Millipore Ltd., Cork, Ireland).

#### 2.8.2. Liquid Chromatography-Mass Spectrometry (LC-MS/MS) Analysis

LC-MS/MS analysis was performed by an UltiMate 3000 RSLC nano-high-performance liquid chromatography system (Thermo Fisher Scientific, Waltham, MA, USA) coupled with a high-resolution Orbitrap Fusion Lumos Tribrid Mass Spectrometer (Thermo Fisher Scientific) equipped with a Nanospray Flex source (Thermo Fisher Scientific). Specifically, Tryptic peptides (0. 5 μg) from each digested sample were loaded into a PepMap RSLC C18 column (2 μm, 100 Å, 50 μm × 15 Cm; Thermo Scientific.) maintained at 40 °C and then separated by a 155 min reversed-phase gradient at 0.3 µL/min flow rate. Eluent A consisted of an aqueous solution of 0.1% formic acid (FA), while eluent B was acetonitrile with 0.1% FA. The gradient program was set as follows: 3% B and 97% A (min 0–110), 20% B and 80% A (min 110–120), 40% B and 60% A (min 120–125), 90% B and 10% A (min 125–145), and 3% B and 97% A (min 145–155). Then the separated peptides were analyzed using Orbitrap Fusion Lumos Tribrid in positive ionization mode (1800 V) with fragmentation in Higher-energy Collision Dissociation (HCD) cell and in Data-Dependent Acquisition mode. Each pooled sample was analyzed in technical triplicate.

#### 2.8.3. Protein Identification and Label-Free Quantification

MS raw data were analyzed with the software PEAKS (v.XPro, Bioinformatics Solutions Inc.). The qualitative identification of proteins was obtained by searching a curated database encompassing *L. rhamnosus* (Taxon ID: 47715) in addition to *Origanum* vulgare (Taxon ID: 39352) database, (UniProtKB/Swiss-Prot Knowledge databases). The search parameters used were set as follows: parent mass error tolerance automatic 10.0 ppm; fragment mass error tolerance 0.6 Da; minimum of 3 fragment ions matched per protein; minimum of 3 peptides matched per protein; 1 missed cleavage; carbamidomethylation of cysteines and oxidation of methionine as fixed and variable modifications; and false discovery rate (FDR) of the identification algorithm lower than 1%. The protein abundance analysis was performed using the label-free quantification (LFQ) module implemented in PEAKS Studio, based on the technical replicates obtained for each pooled experimental condition. Since the technical replicates represent repeated analytical measurements of the same pooled biological sample, they were not considered independent biological replicates for statistical inference. Proteins showing a relative abundance change of at least 20% between conditions were considered modulated. Given the absence of independent biological replicates after pooling, no inferential statistical analysis of biological differences between conditions was performed, and the proteomic analysis was considered exploratory. Proteins showing a relative abundance change of at least 20% between conditions were considered modulated.

Given the absence of independent biological replicates after pooling, no inferential statistical analysis of biological differences between conditions was performed, and the proteomic analysis was considered exploratory.

#### 2.8.4. Pathway Analysis

The molecular functions, biological processes and cellular compartments of the differentially significant expressed proteins were investigated using the Unipept software (2.0.0 v.). Figures were made with the ggplot2 library in R (version 3.5.1).

### 2.9. Cell Culture

Caco-2 cells from passage 5 to 20 were cultured in 25 cm^2^ flasks (Corning Inc. Life Sciences, Tewksbury, MA, USA) using Dulbecco’s modified Eagle’s medium (DMEM, Gibco, Thermo Fisher Scientific, USA) supplemented with 20% fetal bovine serum (FBS, Gibco, Thermo Fisher Scientific, USA), 1% L-glutamine 200 mM (Gibco, Thermo Fisher Scientific, USA), 1% penicillin-streptomycin 10,000 U/mL (Gibco, Thermo Fisher Scientific, USA) and maintained at 37 °C under 5% CO2. Cells were seeded at 104 cm^2^ in 96-well plates (Corning Inc. Life Sciences, USA) for cytotoxicity assay.

### 2.10. Cell Viability

Cytotoxicity was assessed by performing alamarBlue (Thermo Fisher Scientific, USA) assay. Cells were treated when they reached 80–90% of confluence. Adherent cells were treated with decreasing concentrations from 1% *v*/*v* to 0.0156% *v*/*v* of EOs (Tween 80 at the same percentage of EOs was utilized as emulsifier) and from 50% *v*/*v* to 0.37% *v*/*v* of Hys for 24 h. Then, they were washed with phosphate-buffered saline (Gibco, Thermo Fisher Scientific, USA) and incubated for 3 h with 10% (*v*/*v*) of AlamarBlue reagent diluted in culture medium. Fluorescence (RFU or relative fluorescence units) was detected by 560 nm excitation and 590 nm emission, with Cytation 5 Cell Imaging Multi-Mode Reader (Agilent Technologies, Santa Clara, CA, USA). Untreated cells were used as positive controls for 100% viability. Three different experiments were performed in triplicate and the cell viability was calculated according to the following equation:

$$\%\;\mathrm{viability}=\frac{\mathrm{Treated cells}\;(\mathrm{RFU})}{\mathrm{Untreated cells}\;(\mathrm{RFU})}\times 100$$

### 2.11. Statistical Analysis

Data are presented as mean ± standard deviation (SD) unless otherwise stated. Technical replicates were averaged within each independent experiment, and independent biological experiments were considered as the experimental unit for statistical analysis. MIC and MBC values were obtained from serial two-fold dilution assays when possible. Values exceeding the highest concentration tested (>50% *v*/*v*) were considered right-censored and were not included in the inferential statistical analysis. Statistical comparisons were performed using two-way ANOVA, considering product type and/or geographical origin as experimental factors, as appropriate, followed by Šídák’s multiple-comparisons test. Microbial growth curves were analyzed by two-way repeated-measures ANOVA, considering treatment and time as factors, followed by Šídák’s multiple-comparisons test to compare treated and untreated cultures at the corresponding time points. Caco-2 cell viability data were analyzed by two-way ANOVA followed by Šídák’s multiple-comparisons test, comparing each HY concentration with the corresponding untreated control. Statistical analyses were performed using GraphPad Prism [version 11.1.0 218] (GraphPad Software, Boston, MA, USA). Statistical significance was defined as *p* < 0.05 and indicated as follows: * *p* < 0.05, ** *p* < 0.005, *** *p* < 0.001, and **** *p* < 0.0005.

Regarding the proteomic analysis, for each experimental condition, cell pellets obtained from three independent experiments were pooled prior to protein extraction, resulting in one pooled biological sample per condition (*n* = 1 at the biological level). Each pooled sample was analyzed by LC-MS/MS in three technical replicates. Label-free quantification was performed using the PEAKS Studio LFQ module. Since the technical replicates do not represent independent biological replicates, no inferential statistical analysis of biological differences between conditions was performed. Proteins showing a relative abundance change of at least 20% between conditions were considered modulated, and the proteomic results are therefore presented as exploratory.

## 3. Results

### 3.1. Cultivation of the Plant Material

The cultivation of OH plants in the two experimental fields, in the farm “Azienda Agricola San Germano” (Potenza) and in “Il Giardino delle erbe” (Ravenna), lasted 149 days. Plants grew healthily, without any of them showing signs of phytopathology, and were harvested in excellent condition. From both cultivations, approximately 0.8 kg of fresh plant material was obtained, which was then sent for distillation.

### 3.2. Distillation

The yields of Hys and EOs obtained from the distillation are summarized in Table 1.

### 3.3. GC/MS Analysis

Table 2 lists the 41 chemical compounds highlighted by gas-chromatography analyses. The number of compounds is clearly higher in EOs than in Hys. The two EOs have an almost overlapping chemical profile; carvacrol is the main component (78.1% in Lucan EO; 71.1% in Casola EO) and the only detectable quantitative difference concerns the percentage content of p-cymene which is higher in Casola EO (6.5%) than in Lucan EO (2.9%). Regarding the Hys, in Casola Hy 17 components were detected, while in Lucano Hy only five components. Carvacrol was the most abundant component covering percentage values higher than 90%. The percentage values reported in the table represent relative quantities calculated via peak area normalization from GC-FID chromatograms and expressed as a percentage of the total composition.

### 3.4. Antibacterial Activity of OH-EO

The MIC values ± standard deviations (St. Dev.) of OH-EO and OH-HY obtained from Casola Valsenio (Casola-EO) and Lucania (Lucan-EO) against *E. coli* and *L. rhamnosus* strains were tested. Data are summarized in Table 3. The two EOs exhibit the same antimicrobial activity, being more active on the pathogenic strain than the probiotic one. Instead, both Hys show higher MIC values than EOs with apparently greater efficacy on the probiotic strain against the tested strains, while the Hys show inhibitory and cytotoxic effects only against *L. rhamnosus*.

The MBC values and their St. Dev. of OH-EOs and OH-Hys against *E. coli* and *L. rhamnosus* strains are summarized in Table 4. No significant differences are detected either by comparing these values with those obtained studying the inhibitory activity.

To account for the markedly different concentrations of volatile compounds in EOs and Hys, the previously described conversion factor (CF) [9] was applied. The CF was calculated from the ratio between the total volatile abundance (TA) of the EO and that of the corresponding HY and was subsequently used to normalize the Hy MIC values. As shown in Table 5, the normalized MIC_Hy_/CF values were 0.09 for Lucan and 0.19 for Casola, compared with EO MIC values of 0.42 and 0.25, respectively.

### 3.5. Effects of OH-Hys Against L. rhamnosus and E. coli Growth

*L. rhamnosus* treated with both OH-Hys at concentrations of 3–6% *v*/*v* were stimulated both in plateau and exponential phases of its growth curve compared to untreated control, as shown in Figure 1a. After 11 h of incubation, the growth of the probiotic is higher in both treated samples compared to the untreated control, and the growth variation was statistically significant. Moreover, the variation induced by the Casola-Hy showed a higher statistical power (*p* < 0.0005), compared to that induced by the Lucan one (*p* < 0.005). Similarly, in the plateau phase (registered at 17.5 h), both treated samples showed an increase in growth compared to the control. Specifically, the greater growth is achieved in the presence of Lucania-Hy (*p* < 0.0005), while the Casola-Hy induced a lower variation (*p* < 0.005) (Figure 1b).

However, OH-Hys did not affect *E. coli* growth (Figure 2a). It is confirmed by the statistical analysis conducted at the inflection point (11.5 h) and at the end of the incubation (17.5 h). As shown in Figure 2b, no condition significantly stimulates or inhibits the growth of the pathogenic strain compared to the untreated sample.

### 3.6. Proteomic Analysis

In order to evaluate the influence of Hys on the protein profile of the probiotic strain, a preliminary proteomic analysis was conducted to characterize the total proteome of cellular pellets. Specifically, an initial comparison was conducted between the pellets of *L. rhamnosus* treated with Casola-Hy and Lucan-Hy, respectively, assessing the differences in protein abundance compared to the control group (Figure 3).

Label-free analysis revealed a higher relative abundance of specific proteins in the Lucan-Hy condition, particularly enzymes involved in carbohydrate metabolism including glyceraldehyde-3-phosphate dehydrogenase. Delving deeper into the differences between the two treatments, pyruvate dehydrogenase and pyruvate kinase were more abundant in Lucan-Hy compared with the Casola-Hy condition, along with enzymes involved in redox metabolism, including NAD(P)-dependent oxidoreductase and NAD(P)H-binding protein (Supplementary Materials). Conversely, a higher expression of enzymes involved in the pentose phosphate pathway was observed in the Casola-Hy-treated samples, with both phosphopentomutase and formate acetyl-transferase particularly more abundant. The apparent differences in protein abundance of proteins involved in oxidative stress response were also detected, with several proteins showing higher relative abundance in the Lucan-Hy condition. In particular, the universal stress protein and methionyl-tRNA ligase, the latter exhibiting approximately four times higher expression compared to the Casola-Hy treatment, were notably more abundant. Furthermore, increased activity related to cell division and protein synthesis mechanisms was observed. Several proteins, such as the ribosome recycling factor and the cell division protein, exhibited higher relative abundance in the Lucan-Hy treatment. However, a subset of proteins was more abundant in the Casola-Hy hydrolate treatment, possibly indicating differential modulation of proteins associated with these cellular processes.

### 3.7. Cell Viability

Caco-2 cell viability evaluated by the alamarBlue assay in the presence of different concentrations of OH-Hys is reported in Figure 4. The results show that concentrations equal to 50–25% *v*/*v* were cytotoxic, 12.50–6.25% *v*/*v* reduced cell viability while none of concentrations used in the 3.12–0.78% *v*/*v* range affected the cell viability during the incubation period.

## 4. Discussion

The present study compared EOs and Hys obtained from OH cultivated in two distinct Italian regions and revealed differences in their chemical composition and biological activity. Gas chromatographic analysis shows that the two OH-EOs have a higher content of carvacrol than thymol. Carvacrol accounted for 78.1% of the OH-EO obtained from Basilicata and 71.1% of that obtained from Emilia-Romagna. These values are higher than those reported by Amato et al. [5] for OH-EO from Cilento, in which carvacrol accounted for approximately 50% of the volatile fraction, and markedly differ from the thymol-rich profile described by Napoli et al. [1] for Sicilian crops, where carvacrol represented less than 1%. More recently, Yazar et al. [10] characterized an OH-EO with a similarly carvacrol-rich profile, reporting carvacrol as the predominant compound (76.9%), followed by p-cymene (12.84%). This composition is therefore broadly consistent with the carvacrol-rich profiles observed in the present study. Conversely, thymol represented only a minor component of the EOs investigated here (1.3% of the OH-EO obtained from Basilicata and 2.9% of that obtained from Emilia-Romagna), similarly to the low thymol abundance reported by Amato et al. [5] but markedly differing from the thymol-rich profile described by Napoli et al. [1]. Taken together, these findings highlight the considerable chemical variability reported for OH-EOs from different geographical origins. Such variability may reflect the combined influence of climatic and geographical conditions, soil characteristics, and developmental stage. Since the plants cultivated in the two experimental sites originated from the same seed source, variability related to genetic background or ecotype is expected to be limited. However, the individual contribution of the different environmental factors was not specifically investigated in the present study and therefore cannot be conclusively established.

The Hys showed a markedly different volatile profile from the corresponding EOs, despite originating from the same plant material and distillation process. Carvacrol represented 91.3% and 97.5% of the detected volatile fraction in the Emilia-Romagna and Basilicata Hys, respectively, while thymol remained a minor constituent (Emilia-Romagna = 3.5%, Basilicata = 2.2%). Otherwise, all the other essential oil (EO) constituents were not detected, or were detected only at trace levels, in the hydrolate (HY). The distribution of volatile compounds between the oil and aqueous phases depends on several physicochemical properties, including water solubility, volatility, hydrophobicity, oil/water partitioning, and compound-specific behavior during vaporization and condensation. Importantly, the Hy composition was expressed as relative peak-area percentages; therefore, the high relative abundance of carvacrol reflects its proportion within the detected volatile fraction rather than its absolute concentration in the aqueous phase. Similar EO–Hy compositional shifts have been reported for other *Origanum* species. In *O. onites*, carvacrol remained the predominant constituent in both the EO and the corresponding Hy, although its relative abundance and that of several minor terpenes differed between the two fractions [11]. Likewise, in *O. vulgare*, the main chemotypic markers were generally retained in both matrices, but their relative distribution changed substantially, with oxygenated monoterpenes tending to be more represented in the aqueous fraction [12]. These observations support the view that Hy are not simple dilutions of the corresponding EOs, but chemically distinct fractions whose composition reflects compound-specific physicochemical properties and partitioning during distillation.

Nevertheless, despite the differences in the chemical composition of the two EOs and their corresponding HYs, no substantial differences in antimicrobial activity were observed. Indeed, the MIC and MBC values for each type of extract were largely comparable, as shown in Table 3 and Table 4. Specifically, data indicate a greater resistance to EO of the probiotic strain (MIC_Lucan_-EO = 0.420 ± 0.125 and MIC_Casola_-EO = 0.250 ± 0.000) compared to the pathogenic one (MIC_Lucan_-EO = 0.023 ± 0.008 and MIC_Casola_-EO = 0.021 ± 0.008). On the contrary, Hy was found to be more active against the probiotic strain (MIC = 25.000 ± 0.000) compared to the pathogenic one, for which it was not possible to identify a specific MIC (MIC > 50.000). As expected, the MICs of the EOs were lower than those of the Hys, which may partly reflect the higher overall concentration of volatile compounds in the oil phase. Published evidence on the antimicrobial activity of OH-EO remains limited, particularly with regard to OH-Hy. Recent evidence further supports the antimicrobial potential of OH-EO. Yazar et al. [10] reported antimicrobial activity of a carvacrol-rich OH-EO against several bacterial and fungal microorganisms, together with antibiofilm activity. These findings are consistent with the antimicrobial potential observed for the EOs investigated in the present study, although differences in chemical composition, tested microorganisms, and experimental methodologies limit direct quantitative comparisons. Precisely, one study evaluated the antimicrobial activity of OH-EO against *Salmonella Enteriditis*, highlighting the dose-dependent antibiofilm efficacy [13], while a second study evaluated the activity of different EOs against *Pseudomonas putida* isolated from meat. In this study, OH-EO was among the strongest EOs with an MIC =0.05% *v*/*v* [6]. Based on the available literature, few studies have specifically investigated the antimicrobial activity of OH products against microorganisms relevant to the intestinal microbiota, such as *E. coli* (pathogenic strain), and probiotics such as *L. rhamnosus*.

Because EOs and Hys contain markedly different overall amounts of volatile compounds, a direct comparison of their MIC values may underestimate the relative antimicrobial activity of the Hys. After normalization using the previously described conversion factor [9], the MIC_Hy_/CF values remained lower than the corresponding MIC_EO_ values for both extracts (Table 5). This indicates that, to achieve the same antimicrobial activity as the essential oil (EO), a relative concentration of volatiles 4.8 times higher in Casola EO and 1.3 times higher in Lucan EO is required compared to the corresponding hydrosols (Hy). These findings are consistent with our previous results, which support the higher relative efficacy of hydrosols compared to essential oils. Possible explanations for this behavior have been discussed in the literature. For example, Almeida et al. suggested that the hydrophilic nature of hydrolates may favor their diffusion within hydrated extracellular matrices and may contribute to antibiofilm and quorum-sensing-interfering effects [14]. However, these mechanisms were not investigated in the present study and therefore remain speculative in relation to our findings.

Based on the antimicrobial results, the effects of sub-inhibitory Hys concentrations were subsequently investigated in the two bacterial models: the pathogen *E. coli* and the probiotic strain *L. rhamnosus*. Concentrations of 3% and 6% (*v*/*v*) were selected to evaluate bacterial growth under non-inhibitory exposure conditions. The 6% concentration corresponds approximately to the dilution obtained by adding one tablespoon (≈15 mL) of hydrolate to a glass of water (≈250 mL). This concentration was selected to mimic a realistic consumption scenario, as hydrolates are used as ingredients in beverages and functional drinks, and oral administration of diluted hydrolates has been described in the hydrolat therapy literature [15,16,17]. No significant effect on the growth of the tested *E. coli* isolate was detected at the sub-inhibitory Hy concentrations evaluated (Figure 2), whereas both Hys promoted the growth of *L. rhamnosus* under the experimental conditions tested (Figure 1). This effect was characterized by an earlier entry into the exponential growth phase, occurring approximately two hours before that of the untreated control, together with higher optical density (OD) values during the plateau phase, indicating a greater final microbial population density. The increase in plateau-phase OD was more pronounced following exposure to Lucan-Hy than to Casola-Hy. These findings therefore indicate a growth-promoting effect of low Hy concentrations on *L. rhamnosus* in vitro.

These data were further explored by a more targeted investigation of intracellular mechanisms through the study of the total proteome derived from bacterial pellets. The differential expression analysis revealed that *L. rhamnosus* treated with 3% *v*/*v* of Lucan-Hy showed an increased abundance of glyceraldehyde-3-phosphate dehydrogenase, suggesting modulation of carbohydrate metabolism. This enzyme not only plays a central role in carbohydrate metabolism, mainly in glycolysis, suggesting an increased cellular energy demand, but it also contributes significantly to *L. rhamnosus* adhesion to both mucus and intestinal epithelium [18]. Accordingly, the increased abundance of glyceraldehyde-3-phosphate dehydrogenase following Lucan-HY exposure may be relevant to functions beyond glycolysis, although its functional consequences were not directly investigated in the present study. Similarly, the significant expression of the acyl carrier protein suggests an increase in fatty acid biosynthesis in the Lucan-Hy treatment. Furthermore, in Lactobacilli this protein is directly related to the synthesis of fatty acid. Conversely, the reduced expression of cold shock proteins observed in both Hys treatments may be explained by their role in adaptive responses to stressors such as temperature and nutrients [19]. Both HY treatments were associated with reduced abundance of these proteins, which may reflect differences in the adaptive response of bacterial cells to the experimental conditions.

Finally, the total proteome analysis revealed changes consistent with increased representation of proteins involved in carbohydrate metabolism in *L. rhamnosus* treated with Lucan-Hy, as confirmed by the increased expression of key enzymes such as the pyruvate dehydrogenase complex and pyruvate kinase. In contrast, *L. rhamnosus* treated with Casola-Hy was associated with changes in proteins involved in pathways that utilize alternative carbohydrate sources rather than glucose. This is evidenced by the alternative synthesis of acetyl-CoA, which directly enters the Krebs cycle via acetyltransferase expression, as well as the stimulation of the pentose phosphate pathway through phosphopentomutase, which transfers a phosphate group to ribose to produce glycolytic intermediates. These findings may indicate a greater involvement of alternative carbohydrate-processing pathways following Casola-Hy exposure, although the metabolic flux through these pathways was not directly assessed.

Regarding intestinal cell viability, the lower Hys concentrations tested (0.78–3.12% *v*/*v*) did not reduce Caco-2 cell viability after 24 h of exposure (Figure 4). These findings identify a concentration range that was not cytotoxic under the present experimental conditions. However, considering that the 6.25% (*v*/*v*) concentration reduced Caco-2 cell viability, direct use of these hydrolates in food formulations should, pending further investigation, be limited to lower concentrations.

From a sustainability perspective, the characterization of OH-Hys may also contribute to the valorization of a co-product generated during essential oil distillation. Hys are often considered secondary products of the distillation process, although their volatile fraction may retain biologically active compounds of potential interest [6]. Their recovery and characterization may therefore support more efficient use of aromatic plant biomass and contribute to circular-economy approaches aimed at reducing waste and increasing the value of distillation-derived products. Although the present findings do not allow specific applications to be established, the biological properties observed for OH-Hys warrant further investigation of their potential use in food-related, agricultural, or veterinary applications.

In conclusion, to the best of our knowledge, the present study provides the first evidence that *O. heracleoticum* hydrolate may exert a prebiotic-like effect by promoting the growth of probiotic bacteria belonging to the intestinal microbiota. Furthermore, *O. heracleoticum* hydrolate did not exhibit cytotoxic effects on Caco-2 intestinal cells at concentrations ranging from 0.78% to 3.12% (*v*/*v*) after 24 h of exposure. The study has some limitations that should be acknowledged. The antimicrobial activity was evaluated using only one clinical *E. coli* isolate and one *L. rhamnosus* strain; therefore, the observed effects cannot be generalized at the species level and should be confirmed using a broader panel of pathogenic and probiotic strains. In addition, the growth-promoting effect observed on a single probiotic strain is not sufficient to conclusively demonstrate a prebiotic activity. Likewise, the in vitro findings cannot be directly extrapolated to in vivo conditions, where more complex microbial interactions and host-related factors may influence the biological response. Nevertheless, despite its preliminary nature, this study suggests that OH-Hys may exert not only antimicrobial activity but also a selective growth-promoting effect on probiotic bacteria. These findings highlight a previously unexplored biological property of this hydrolate and provide a rationale for future investigations involving additional microbial strains, complex microbial communities, and in vivo models.

## 5. Conclusions

This study evaluated the chemical profile and the antimicrobial activity of EO and Hy extracted from OH grown in two different Italian regions. The chemical profile obtained shows the preponderance of carvacrol compared to thymol, making these extracts comparable to those extracted from OH grown in Cilento rather than in Sicily. This study provides preliminary evidence of a growth-promoting effect of low OH-Hy concentrations on *L. rhamnosus* in vitro, supporting further investigation of their prebiotic potential. Furthermore, these concentrations did not show effects on the growth of a pathogenic strain, such as *E. coli,* nor on the toxicity of intestinal mucosal cells. However, these findings are derived from simplified in vitro models and should therefore be interpreted as preliminary. Further studies involving multiple probiotic strains, complex microbial communities, and in vivo models are required to determine whether these in vitro effects translate into genuine prebiotic activity within the intestinal microbiota and under physiological gastrointestinal conditions, and whether OH-Hys may have future applications in microbiota-oriented or dietary strategies. Although further studies are needed to address the limitations of the present experimental approach, the overall findings identify OH-HYs as chemically distinct distillation products endowed with measurable antimicrobial activity and growth-modulating effects on *L. rhamnosus* in vitro. These results provide a valuable basis for future investigations using broader microbial panels, more complex microbiota models, and in vivo systems.

## Figures and Tables

**Figure 1:**
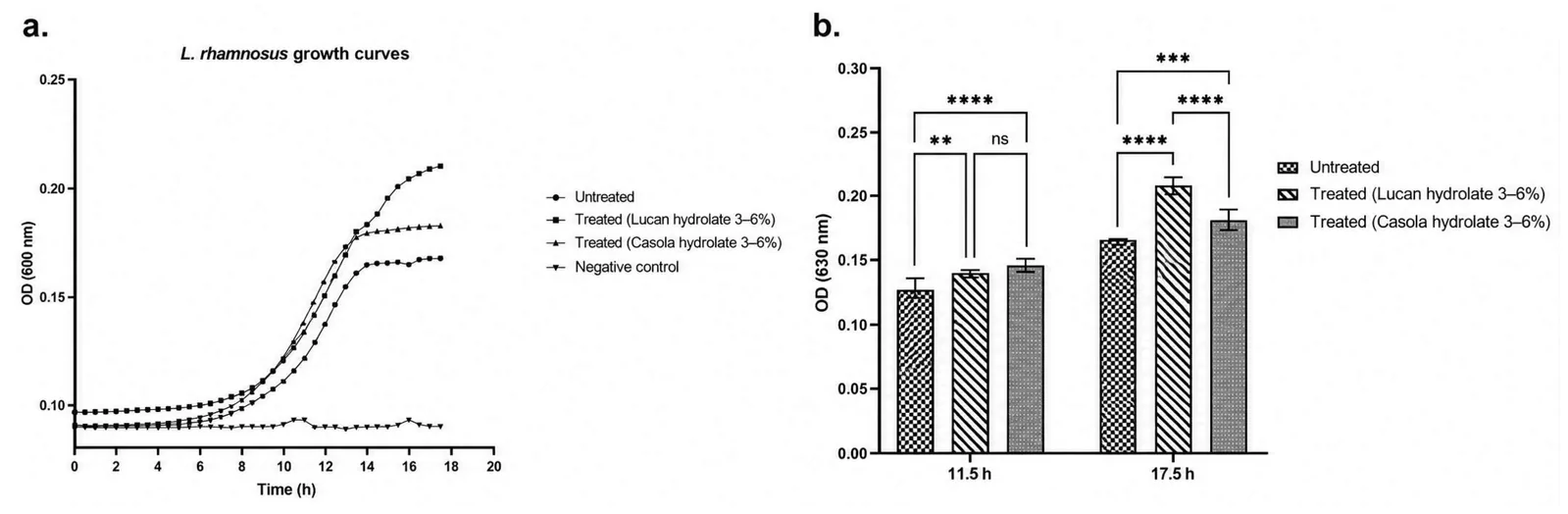
(**a**). Growth curves of *L. rhamnosus* treated with Hys 3% *v*/*v* and 6% *v*/*v*. (**b**). Statistical analysis of the growth of *L. rhamnosus* measured at the inflection point (11.5 h) of the growth curve and in the plateau phase (17.5 h), **** = *p* < 0.0005; *** = *p* < 0.001; ** = *p* < 0.005; ns = not significant.

**Figure 2:**
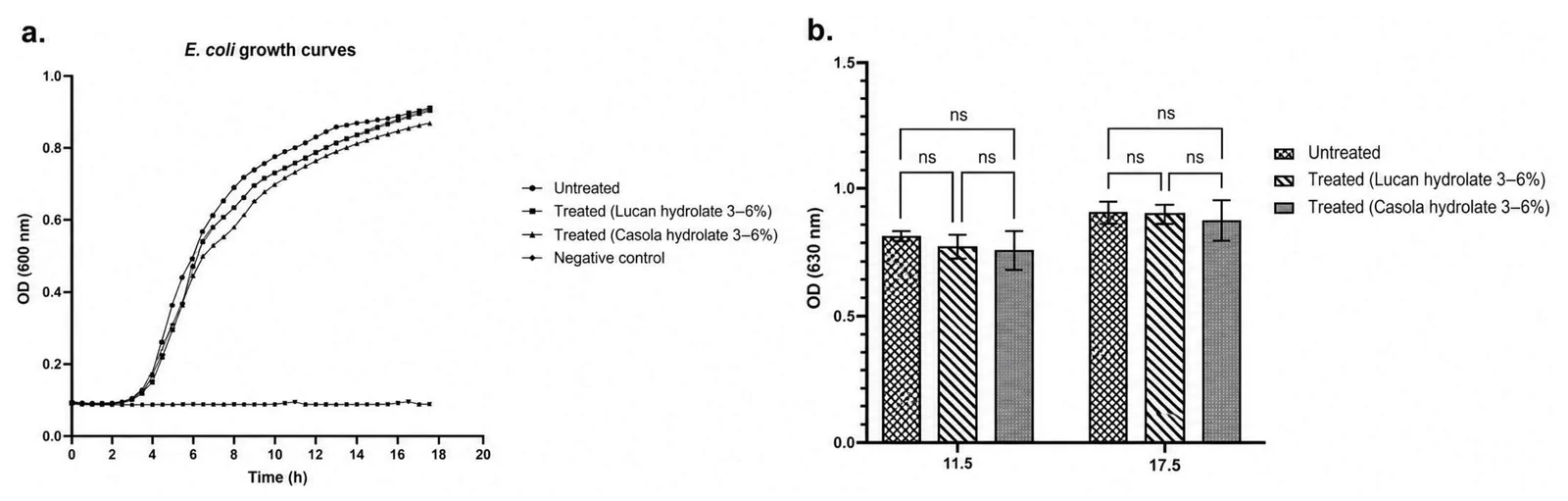
(**a**). Growth curves of *E. coli* treated with Hys 3% *v*/*v* and 6% *v*/*v*. (**b**). Statistical analysis of the growth of *E. coli* measured at the inflection point (11.5 h) of the growth curve and in the plateau phase (17.5 h), ns = not significant.

**Figure 3:**
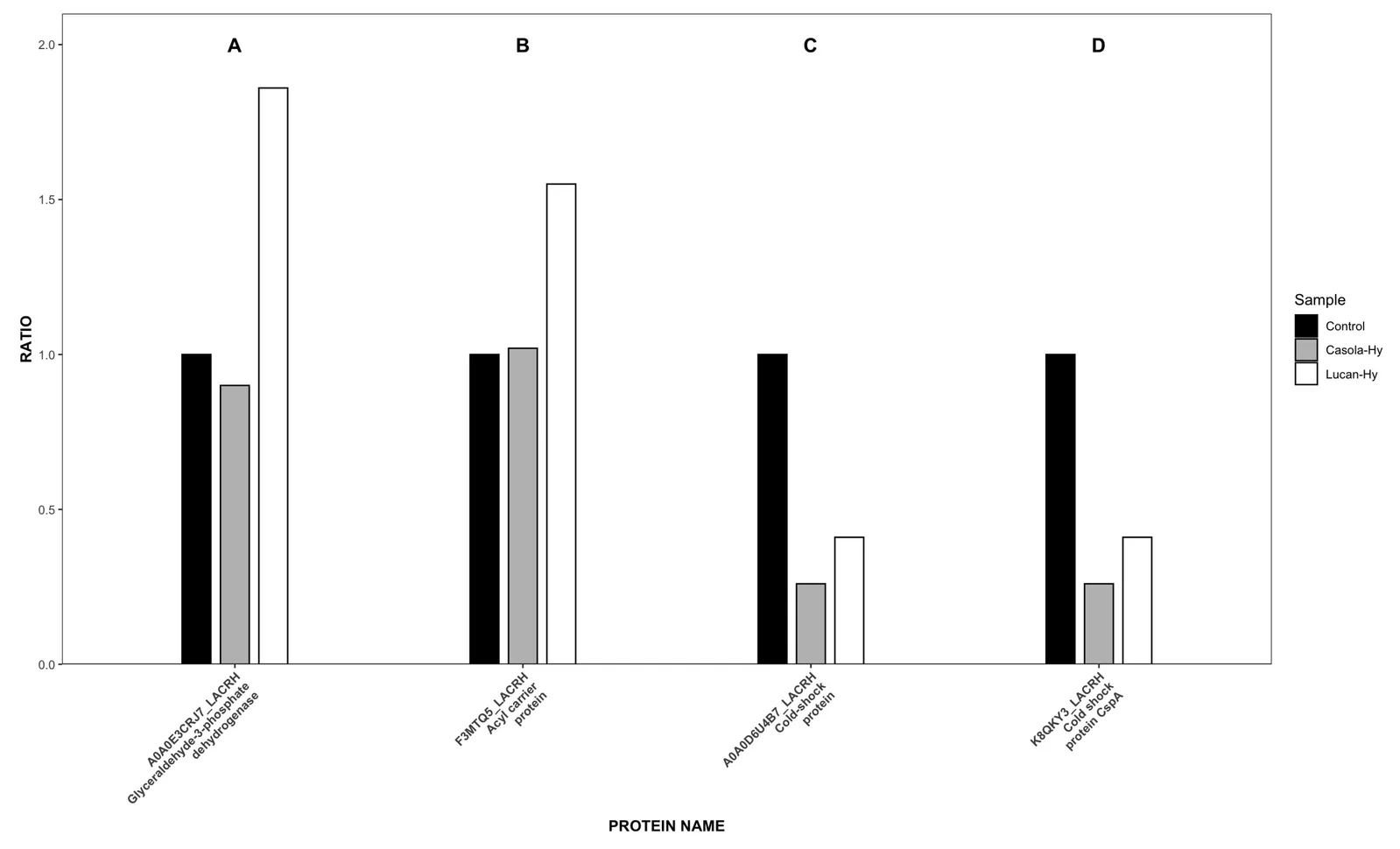
Relative abundance of selected modulated proteins in the two conditions, 3% (*v*/*v*) Casola-Hy and 3% (*v*/*v*) Lucan-Hy, compared with the control, as determined by label-free quantification using PEAKS software. (**A**) A0A0E3CRJ7 (K8QCL6): Glyceraldehyde-3-phosphate dehydrogenase; (**B**) F3MTQ5: Acyl carrier protein; (**C**) A0A0D6U4B7: Cold shock protein; (**D**) K8QKY3: Cold shock protein CspA; LACRH: *L. rhamnosus* (species). Samples represent a single biological sample pooled by condition and analyzed in technical triplicate.

**Figure 4:**
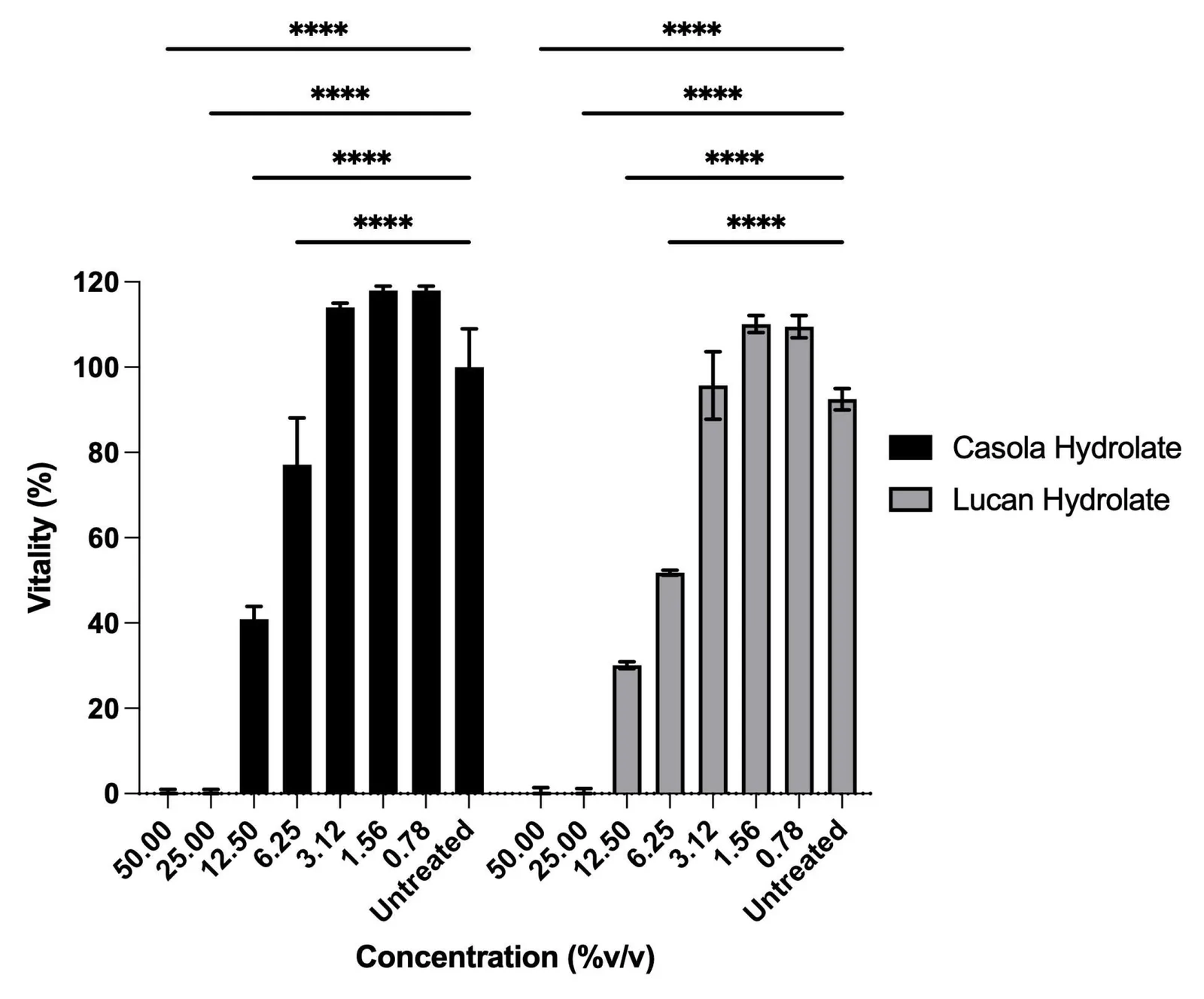
Effect of Casola and Lucan Hys on intestinal cell viability. Caco-2 cell viability was evaluated following treatment with increasing concentrations (0.78–50% *v*/*v*) of Casola Hy (black bars) or Lucan Hy (gray bars). Untreated cells were used as controls. Data are expressed as mean ± St. Dev. from three independent biological experiments, each performed in three technical replicates. Statistical analysis was performed using two-way ANOVA followed by Šídák’s multiple-comparisons test, comparing each treatment concentration with the corresponding untreated control. **** *p* < 0.0001.

**Table 1:** Volumes, extraction yield of EOs, and recovery rates of Hys obtained by distillation.

	Lucan EO	Casola EO	Lucan Hy	Casola Hy
Obtained volume (mL)	2.0	2.5	100	100
Yield (% *v*/*w*)	0.25	0.31	-	-
Recovery ratio (mL/kg FW)	-	-	125	125

**Table 2:** Chemical volatile composition (percentage mean value) of EOs and Hys.

N°	COMPONENT ^1^	LRI ^2^	LRI ^3^	Lucan EO	Casola EO	Lucan Hy	Casola Hy
1	α-thujene	900	902	0.2	0.3	-	-
2	α-pinene	931	928	0.1	0.1	-	-
3	camphene	958	961	0.1	0.1	-	-
4	1-octen-3-ol	982	979	0.9	2.0	0.1	1.9
5	β-pinene	985	980	0.1	-	-	-
6	β-myrcene	990	992	0.7	0.8	-	-
7	α-phellandrene	999	998	0.1	0.1	-	Tr
8	p-cymene	1010	1016	2.9	6.5	-	0.2
9	limonene	1019	1022	0.1	0.1	-	-
10	trans-β-ocimene	1030	1035	0.3	Tr	-	-
11	β-terpinene	1037	1036	-	-	-	0.1
12	cis-β-ocimene	1040	1037	0.1	-	-	-
13	trans-sabinene hydrate	1049	1052	0.3	1.1	-	1.1
14	γ-terpinene	1060	1065	5.3	4.2	-	Tr
15	linalool	1078	1081	0.6	-	-	-
16	terpinolene	1079	1085	0.4	0.5	-	0.3
17	cis-sabinene hydrate	1099	1098	Tr	-	-	Tr
18	3-carene	1138	1142	0.1	-	-	-
19	endo-borneol	1150	1149	1.2	0.7	-	0.6
20	terpinene-4-ol	1159	1161	-	1.5	0.1	-
21	α-terpineol	1170	1174	-	0.3	-	0.1
22	trans-dihydrocarvone	1180	1186	-	0.1	-	-
23	methylthymol	1210	1215	0.2	Tr	0.1	0.1
24	methylcarvacrol	1228	1224	0.8	2.1	-	-
25	thymoquinone	1220	1216	-	0.2	-	-
26	thymol	1270	1266	1.3	2.9	2.2	3.5
27	carvacrol	1285	1278	78.1	71.1	97.5	91.3
28	4-terpinenyl acetate	1289	1286	-	-	-	0.7
29	carvacrol acetate	1350	1354	Tr	0.1	-	Tr
30	(-)-β-bourbonene	1391	1388	Tr	0.1	-	-
31	β-caryophyllene	1420	1424	2.5	2.1	-	Tr
32	β-copaene	1429	1426	0.4	0.3	-	-
33	humulene	1476	1473	0.3	-	-	-
34	β-bisabolene	1498	1495	2.0	2.1	-	Tr
35	α-farnesene	1500	1496	0.1	Tr	-	-
36	germacrene D	1505	1500	0.1	Tr	-	-
38	δ-cadinene	1519	1515	-	0.1	-	-
39	caryophyllene oxide	1575	1580	0.3	0.1	-	-
40	spathulenol	1610	1601	0.1	Tr	-	-
41	α-bisabolol	1669	1665	0.3	-	-	-
	SUM			100.0	99.6	100.0	99.9

^1^ The components are reported according to their elution order on apolar column; ^2^ Linear Retention Indices measured on apolar column; ^3^ Linear Retention Indices from the literature; -: not detected; Tr: percentage mean values ˂ 0.1%.

**Table 3:** OH MIC values. Data are expressed as mean ± St. Dev. from three independent biological experiments, each performed in three technical replicates.

MIC (% *v*/*v* ± St. Dev.)				
	Lucan EO	Casola EO	Lucan HY	Casola HY
*L. rhamnosus*	0.420 ± 0.125	0.250 ± 0.000	25.000 ± 0.000	25.000 ± 0.000
*E. coli*	0.023 ± 0.008	0.021 ± 0.008	>50.000	>50.000

**Table 4:** OH MBC values. Data are expressed as mean ± St. Dev. from three independent biological experiments, each performed in three technical replicates.

MBC (% *v*/*v* ± St. Dev.)				
	Lucan EO	Casola EO	Lucan Hy	Casola Hy
*L. rhamnosus*	0.500 ± 0.274	0.250 ± 0.000	25.000 ± 0.000	25.000 ± 0.000
*E. coli*	0.060 ± 0.000	0.060 ± 0.008	>50.000	>50.000

**Table 5:** Comparison of the antimicrobial activity of OH-EOs and OH-Hys against *L. rhamnosus* after normalization for differences in total volatile abundance (TA).

	Lucan	Casola
EOTA	2.0 × 10^12^	4.9 × 10^11^
HyTA	7.0 × 10^9^	3.8 × 10^9^
CF	2.88 × 10^2^	1.30 × 10^2^
MIC_Hy_/CF	0.09	0.19
MIC_EO_	0.42	0.25

Note. EOTA and HYTA indicate the total volatile abundance measured in the corresponding EO and Hy, respectively. The conversion factor (CF) was calculated as the ratio between EOTA and HYTA and was used to normalize the HY minimum inhibitory concentration (MIC). MIC_Hy_/CF represents the HY MIC adjusted for the difference in volatile abundance, whereas MIC_EO_ represents the experimentally determined MIC of the corresponding EO.

## Data Availability

The raw data supporting the conclusions of this article will be made available by the authors on request.

## Supplementary Materials

The following supporting information can be downloaded at https://www.mdpi.com/article/10.3390/biology15201857/s1. Table S1. Enriched metabolic pathways (*p* < 0.05) identified through the analysis of proteins expressed under the two conditions (IL vs IC), along with the corresponding protein expression ratios.

## References

[1] Napoli E., Giovino A., Carrubba A., How Yuen Siong V., Rinoldo C., Nina O., Ruberto G. (2020). Variations of Essential Oil Constituents in Oregano (*Origanum vulgare* Subsp. Viridulum (=*O. heracleoticum*) over Cultivation Cycles. Plants.

[2] Della Pepa T., Elshafie H.S., Capasso R., De Feo V., Camele I., Nazzaro F., Scognamiglio M.R., Caputo L. (2019). Antimicrobial and Phytotoxic Activity of *Origanum heracleoticum* and *O. majorana* Essential Oils Growing in Cilento (Southern Italy). Molecules.

[3] Conforti F., Marrelli M., Menichini F., Tundis R., Statti G.A., Solimene U., Menichini F. (2011). Chemical Composition and Protective Effect of Oregano (*Origanum heracleoticum* L.) Ethanolic Extract on Oxidative Damage and on Inhibition of NO in LPS-Stimulated RAW 264.7 Macrophages. J. Enzym. Inhib. Med. Chem..

[4] Salerno G., Guarrera P.M., Caneva G. (2005). Agricultural, Domestic and Handicraft Folk Uses of Plants in the Tyrrhenian Sector of Basilicata (Italy). J. Ethnobiol. Ethnomed..

[5] Amato G., Caputo L., Francolino R., Martino M., De Feo V., De Martino L. (2023). *Origanum heracleoticum* Essential Oils: Chemical Composition, Phytotoxic and Alpha-Amylase Inhibitory Activities. Plants.

[6] Oussalah M., Caillet S., Saucier L., Lacroix M. (2006). Antimicrobial Effects of Selected Plant Essential Oils on the Growth of a *Pseudomonas putida* Strain Isolated from Meat. Meat Sci..

[7] Sienkiewicz M., Wasiela M., Głowacka A. (2012). The antibacterial activity of oregano essential oil (*Origanum heracleoticum* L.) against clinical strains of *Escherichia coli* and *Pseudomonas aeruginosa*. Med. Dosw. Mikrobiol..

[8] D’Amato S., Serio A., López C.C., Paparella A. (2018). Hydrosols: Biological Activity and Potential as Antimicrobials for Food Applications. Food Control.

[9] Di Vito M., Smolka A., Proto M.R., Barbanti L., Gelmini F., Napoli E., Bellardi M.G., Mattarelli P., Beretta G., Sanguinetti M. (2021). Is the Antimicrobial Activity of Hydrolates Lower than That of Essential Oils?. Antibiotics.

[10] Yazar S., Beyzi E., Stankov S., Çağlayan M., Burcu Külahcı M., Stoyanova A., Yilmaz S. (2025). Bioactive Potential of *Origanum heracleoticum* L. Essential Oil: Chemical Composition and Its Antimicrobial and Anticancer Properties. J. Essent. Oil Bear. Plants.

[11] Özliman S. (2026). Volatile Compounds of Volatile Oils and Hydrosols: Intra-Specific Comparison in Seven Lamiaceae Species. BMC Plant Biol..

[12] Freire C.S., Cardoso M.D.G., Póvoa O., Farinha N., Nelson D.L., Machado A.M., Figueiredo A.C. (2025). Comparative Evaluation of the Volatile Profile of the Essential Oil, the Hydrolate and the Plant Material from *Origanum vulgare* Subsp. Virens Grown in Portugal. Foods.

[13] Čabarkapa I., Čolović R., Đuragić O., Popović S., Kokić B., Milanov D., Pezo L. (2019). Anti-Biofilm Activities of Essential Oils Rich in Carvacrol and Thymol against *Salmonella* Enteritidis. Biofouling.

[14] Almeida H.H.S., Fernandes I.P., Amaral J.S., Rodrigues A.E., Barreiro M.-F. (2024). Unlocking the Potential of Hydrosols: Transforming Essential Oil Byproducts into Valuable Resources. Molecules.

[15] Aćimović M., Tešević V., Smiljanić K., Cvetković M., Stanković J., Kiprovski B., Sikora V. (2020). Hydrolates: By-Products of Essential Oil Distillation: Chemical Composition, Biological Activity and Potential Uses. Adv. Technol..

[16] Tavares C.S., Gameiro J.A., Roseiro L.B., Figueiredo A.C. (2022). Hydrolates: A Review on Their Volatiles Composition, Biological Properties and Potential Uses. Phytochem. Rev..

[17] Catty S. (2001). Hydrosols: The Next Aromatherapy.

[18] Deng Z., Dai T., Zhang W., Zhu J., Luo X., Fu D., Liu J., Wang H. (2020). Glyceraldehyde-3-Phosphate Dehydrogenase Increases the Adhesion of *Lactobacillus reuteri* to Host Mucin to Enhance Probiotic Effects. Int. J. Mol. Sci..

[19] Sabaitis J.E., Powell G.L. (1976). Acyl Carrier Protein Metabolism and Regulation of Fatty Acid Biosynthesis by *Lactobacillus plantarum*. J. Biol. Chem..

