# Supplementary material for: In Vitro Study of the Antimicrobial and Prebiotic Activity of Origanum heracleoticum Hydrolates from Two Italian Crops and the Proteomic Response of Probiotic Bacteria
Source: Biology (Basel). 2026 Oct 15;15(20):1857. doi: 10.3390/biology15201857 (PMC13701891; doi:10.3390/biology15201857)
Supplement: Supplementary file 1 [file biology-15-01857-s001.zip › biology-4574283-supplementary.pdf]

Supplementary Table

Table S1. Enriched metabolic pathways ($p < 0.05$) identified through the analysis of proteins expressed under the two conditions (IL vs IC), along with the corresponding protein expression ratios.

PROTEIN ACCESSION	PROTEIN DESCRIPTION	Lucan-Hy *vs* Casola-Hy ratio
**Cell division**		
A0A2A5L7A9	30S ribosomal protein S2	1,33
A0A7S7FRG9	50S ribosomal protein L33	1,41
A0A0D6U733	DNA-directed RNA polymerase subunit alpha	1,29
A0A0D6UBJ9	Elongation factor Tu	1,27
A0A0E3CQ94	Universal stress protein	1,61
A0A180ABC8	Methionine--tRNA ligase	4,4
A0A0E3CRJ2	Ribosome-recycling factor	1,86
F3MUW6	30S ribosomal protein S3	-2,08
A0A508Z3R2	Cell division protein FtsZ	1,55
A0A0E3CRF6	Uridylate kinase	1,63
A0A809MST9	Favin-nucleotide-binding protein	1,43
A0A171J992	Trigger factor	-1,54
A0A807RP89	Endoribonuclease YbeY	1,46
A0A7S7JHD5	30S ribosomal protein S10	1,38
A0A0H0YRV0	Translation initiation factor IF-1	-1,25
A0A873ZHH6	50S ribosomal protein L15	-1,23
**Stress Response**		
A0A2A5L7W8	Chaperonin GroEL	1,52
A0A508YYN2	FAD-dependent oxidoreductase	1,47
A0A508YQE1	Peroxiredoxin	1,4
A0A7S7FPM3	Thioredoxin	1,43
A0A0D6UB62	Cysteine desulfurase	1,61
**Glucose Metabolism**		
A0A508YZT9	Alpha-ketoacid dehydrogenase subunit beta	1,24
A0A171JAF8	Enolase	1,32
A0A249DFR4	L-lactate dehydrogenase	1,51
A0A7S7FNT6	Pyruvate dehydrogenase E1 component subunit alpha	1,7
A0A5P5Z8J8	Glycoside hydrolase	-1,30
A0A0D6UD09	Phosphocarrier protein HPr	-1,54
A0A0H0YQH2	Formate acetyltransferase	-2,27
A0A171J9P8	ATP synthase subunit beta	1,38
A0A249DBA7	Pyruvate kinase	1,63
A0A0E3CRJ2	6-phosphogluconate dehydrogenase decarboxylating	1,28
A0A0J7A425	2 3-bisphosphoglycerate-dependent phosphoglycerate mutase	1,19
A0A807RRB1	NAD(P)-dependent oxidoreductase	1,62
A0A2A5L811	NAD(P)H-binding protein	1,62
F3N0L9	Flavodoxin	1,41

A0A807RP89	Aldo/keto reductase	1,46
A0A249DE53	Phosphoenolpyruvate-protein phosphotransferase	1,6
A0A7Z9DRM1	Dihydrolipoamide acetyltransferase component of pyruvate dehydrogenase complex	1,56
**Non-glucose carbohydrate metabolism**		
A0A7S7FNQ5	Galactokinase	-1,16
A0A508YIY0	Ribose-5-phosphate isomerase A	1,49
A0A0D6U4Z4	Ribose-phosphate pyrophosphokinase	-1,10
K8QTI6	Phosphoenolpyruvate-dependent sugar phosphotransferase system EIIA putative mannose specific	-1,05
A0A0E3CRT4	PTS fructose transporter subunit IIA	-1,05
A0A5P5ZBV1	Adenine phosphoribosyltransferase	-1,32
A0A873ZPJ2	L-lactate oxidase	1,76
K8QTG4	Phosphopentomutase	-3,70
A0A7S7FRS9	UTP--glucose-1-phosphate uridylyltransferase	-1,29
**Protein metabolism**		
A0A839HVM9	Asparagine synthetase B	2,18
A0A0E3CNH0	Leucine--tRNA ligase	-1,11
A0A249DH01	Arginine--tRNA ligase	1,33
**Lipid metabolism**		
A0A179ZXH5	Beta-ketoacyl-[acyl-carrier-protein] synthase III	3,14
F3MTQ5	Acyl carrier protein	1,31
A0A7S7FP24	Glycerol kinase	1,6
A0A7S7JH89	acetyl-CoA carboxytransferase	1,91
A0A508YUM4	Dihydrolipoyl dehydrogenase	-1,08
A0A249DDZ9	lipoate--protein ligase	1,42
**Metal transporters**		
A0A508Z3R2	Metal ABC transporter ATP-binding protein	1,55
A0A249DE53	Manganese ABC transporter ATP-binding protein	1,6
A0A873ZKN9	Iron chelate uptake ABC transporter family permease subunit	1,55
A0A7S7JGS8	Manganese ABC transporter inner membrane permease protein SitD	1,45
A0A7Z9DRM1	ATP-dependent zinc metalloprotease FtsH	1,56
A0A249DDZ9	Manganese ABC transporter substrate-binding protein	1,42
A0A7S7JHD5	Zinc ABC transporter substrate-binding protein	1,38
A0A873ZKN9	ATP-dependent zinc metalloprotease FtsH	1,55
